# A Case Report and Literature Review of New-Onset Myasthenia Gravis After COVID-19 Infection

**DOI:** 10.7759/cureus.33048

**Published:** 2022-12-28

**Authors:** Tulika Chatterjee, Sriviji Senthil Kumaran, Moni Roy

**Affiliations:** 1 Internal Medicine, University of Illinois, Peoria, USA

**Keywords:** neuromuscular diseases, neuromuscular, anti-acetylcholine receptor antibody, myasthenia gravis, post-infectious myasthenia gravis

## Abstract

Myasthenia gravis (MG) is an autoimmune disorder affecting the neuromuscular junction caused by a B-cell-mediated, T-cell-dependent immunologic attack at the end plate of the postsynaptic membrane. Attack on muscle acetylcholine receptors (AChR) of the postsynaptic membrane due to the AChR, muscle-specific tyrosine kinase, or lipoprotein receptor-related peptide 4 antibodies lead to symptoms of painless, fluctuating weakness of muscle groups and often begins with ocular signs and symptoms. Coronavirus disease 2019 (COVID-19) is an acute respiratory disease caused by severe acute respiratory syndrome coronavirus 2 (SARS-CoV-2), a novel coronavirus closely related to SARS-CoV. Serious neurologic complications are infrequent and diverse with reported cases of stroke, encephalitis/meningitis, Guillain-Barré syndrome, acute disseminated encephalomyelitis, ataxia, and unspecified limb weakness. MG is a rarely reported sequela of COVID-19 infection. To date, there are 15 reported cases of post-COVID-19 MG. In this article, we present a case of post-COVID-19 MG and a concise review of other reported cases. An 83-year-old Caucasian male with a medical history of atrial fibrillation status post-ablation and non-ischemic cardiomyopathy was initially admitted for COVID-19 pneumonia. He was treated with remdesivir, convalescent plasma, and supplemental oxygen therapy but did not require invasive mechanical intubation. One month after discharge, he started experiencing fatigue with muscle weakness and progressive dyspnea. He progressed to develop dysphonia, especially at the end of the day. After extensive workup, he was diagnosed with MG with a positive antibody against the AChR. The chronological events of developing slowly worsening muscular weakness after recovering from COVID-19 infection and positive AChR antibody led to the diagnosis of post-COVID-19 new-onset MG. Post-COVID-19 fatigue, long-term use of steroids, and intensive care unit-related physical deconditioning can be confounders in the clinical presentation of post-COVID-19 new-onset MG. Careful history-taking and meticulous assessment of chronological events are needed to diagnose this rare entity.

## Introduction

As the coronavirus disease 2019 (COVID-19) pandemic has started stabilizing, the secondary complications and long-term sequelae of the severe acute respiratory syndrome coronavirus 2 (SARS-CoV-2) infection have begun to surface [1]. It is evident that the morbidity of SARS-CoV-2 infection extends beyond the phase of acute respiratory illness and may affect any organ besides the respiratory system, including the cardiovascular, hematological, and nervous systems [2]. Dysgeusia, anosmia, chronic headaches, hemorrhagic or ischemic strokes, encephalitis/meningitis, encephalopathy, Guillain-Barré syndrome, acute disseminated encephalomyelitis (ADEM), ataxia, neuropathy, and unspecified limb weakness have been reported as post-COVID-19 neurological complications [3,4]. New-onset myasthenia gravis (MG) is a rarely reported neuromuscular complication of SARS-CoV-2 infection. MG is an autoimmune disorder affecting the neuromuscular junction caused by a B-cell-mediated, T-cell-dependent immunologic attack at the end plate of the postsynaptic membrane [5]. Attack on muscle acetylcholine receptors (AChR) of the postsynaptic membrane due to the acetylcholine AChR, muscle-specific tyrosine kinase (MuSK), or lipoprotein receptor-related peptide 4 (LRP4) antibodies lead to symptoms of painless, fluctuating weakness of muscle groups and often begins with ocular signs and symptoms [3].

Diagnosis of MG may be difficult in the post-infectious phase of COVID-19. Symptoms of chronic fatigue and generalized muscular weakness are frequently seen as a part of the long COVID-19 sequela. Furthermore, prolonged duration of hospital stays, mechanical ventilation, limited mobility with isolation, and steroid use can contribute to neuromuscular weakness. A detailed history and a precise time frame of symptom onset play a role in raising suspicion for MG. The literature review showed only 15 reported cases of post-COVID-19 new-onset MG. We present a case of post-COVID-19 new-onset MG in an elderly male with no previous history of neuromuscular or autoimmune disorder.

## Case presentation

**Figure 1:**
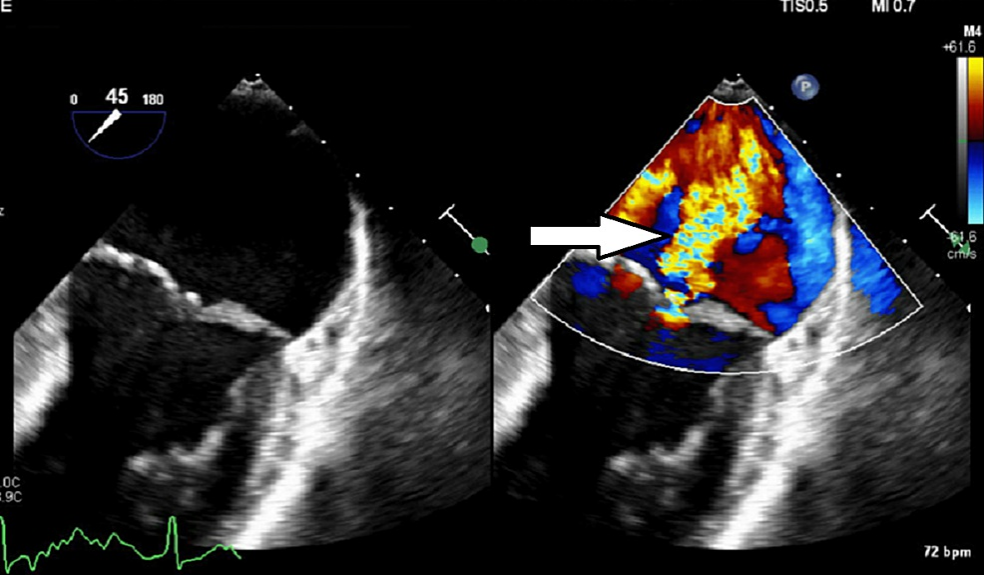
Transesophageal echocardiogram, mid-esophageal mitral commissural view showing severe mitral regurgitation (right arrow).

**Figure 2:**
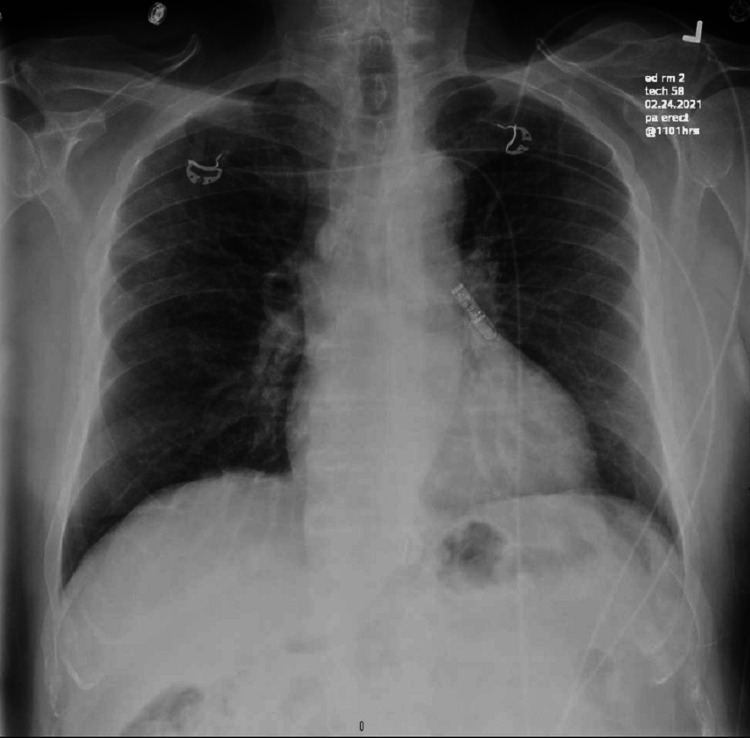
Chest X-ray showing ill-defined opacities over bilateral upper lobe with no consolidation.

**Figure 3:**
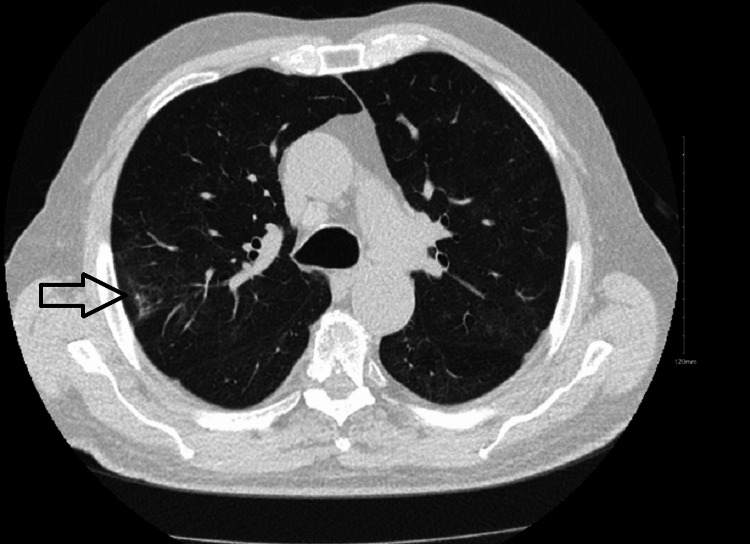
Computed tomography of the chest without contrast showing mild emphysematous changes with prominent interstitial markings in the right lobe (right arrow).

An 83-year-old Caucasian male with a medical history of atrial fibrillation status post-ablation, non-ischemic cardiomyopathy, trigeminal neuralgia, and moderate mitral regurgitation was admitted to the intensive care unit (ICU) in December 2020 with acute respiratory failure due to COVID-19 pneumonia. He was treated with heated high-flow oxygen, remdesivir, and convalescent plasma therapy and was hospitalized for 17 days. He was unvaccinated against SARS-CoV-2 at the time of the initial infection. A month after discharge, he started experiencing significant fatigue with muscle weakness and progressive dyspnea. Workup for these symptoms was initially pursued in February 2021. This included an echocardiogram showing moderate-to-severe mitral regurgitation (Figure 1) and a chest X-ray (Figure 2) which showed ill-defined opacities in the upper lung zones suggestive of atelectasis versus viral pneumonia. A follow-up computed tomography (CT) of the chest showed mild emphysematous changes with prominent interstitial markings in the right upper and middle lobe with no consolidation, pleural effusion, and pulmonary fibrosis (Figure 3). At this time, his dyspnea was attributed to worsening mitral regurgitation. However, his fatigue continued to progress. Due to a history of atrial fibrillation, a loop recorder was implanted which did not show any increased atrial fibrillation burden contributing to his fatigue. His generalized weakness continued to progress over the next few months, and his coworkers reported that his speech would progressively become incomprehensible throughout the day. In September 2021, he was admitted again with acute hypoxemic respiratory failure requiring intubation and mechanical ventilation. He was suspected of having aspiration pneumonia and possibly angiotensin receptor inhibitor-induced angioedema. He was treated with broad-spectrum antibiotics and methylprednisolone 80 mg. Thereafter, methylprednisolone was switched to prednisone 40 mg daily, which was tapered over the course of five days. He was weaned off the ventilator and extubated. After the completion of his taper, his dysarthria and dysphagia worsened. C1 esterase inhibitor function returned normal (result: 83%, normal: >67%).

Post-hospitalization, he was found to have orthostatic hypotension and bilateral fatigable ptosis. Laboratory workup for fatigue is presented in Table 1. Vitamin B12 was found to be at a low normal level at 234 pg/mL, and high-dose replacement was started with no significant clinical improvement. The anti-AChR antibody was elevated at 0.26 nmol/L (reference: <0.02 nmol/L), the anti-MuSK antibody was negative, and AChR modulating antibody flow cytometry was negative. The patient had no personal or family history of neuromuscular or autoimmune disorders.

**Table 1:** Laboratory tests done for evaluation of fatigue. AChR = acetylcholine receptor; MuSK = muscle-specific tyrosine kinase

Laboratory test	Value
Antinuclear antibody screen	Negative
Lyme antibody screen	Negative
Vitamin B12	234 pg/mL (reference: 200–900 pg/mL)
Thyroid-stimulating hormone	1.27 mIU/L (reference: 0.300–5.000 mIU/L)
Anti-AChR antibody	0.26 nmol/L (reference: <0.02 nmol/L)
Anti-MuSK antibody	Negative
AChR modulating antibody flow cytometry	Negative

A brief course of prednisone (40 mg/40 mg/30 mg/30 mg/20 mg/20 mg/10 mg/10 mg over eight days) was started by his primary care provider, and he was referred urgently to a neurologist. Symptoms again improved with prednisone but returned upon completion. His neurologist started him on prednisone 60 mg every other day and pyridostigmine 30 mg daily for residual weakness. However, the patient could not tolerate pyridostigmine, resulting in its discontinuation. The patient was started on azathioprine 50 mg twice daily, and the dose of the steroid was reduced every two weeks. A CT of the chest ruled out underlying thymoma. His symptoms of fatigue improved while on azathioprine treatment.

His MG was well controlled on azathioprine. However, he developed worsening heart failure and a decline in renal function within a year. Approximately one year after his MG diagnosis, he developed a bilateral periprosthetic joint infection with *Streptococcus oralis*/*mitis* bacteremia. He passed away from multiorgan failure.

## Discussion

Post-COVID-19, new-onset neurological autoimmune diseases are being increasingly recognized and reported [6-9]. MG is an autoimmune condition that is caused by autoantibodies directed toward the postsynaptic AChR on neuromuscular junctions [10,11]. Blockade of the AChR on the postsynaptic membrane causes a decrease in action potential generation resulting in muscular weakness. It is a relatively rare disease with a prevalence of 50-125 cases per million [12].

The presentation and prognosis of COVID-19 in patients with pre-existing MG have been published since the onset of the COVID-19 pandemic [13-18]. Both new-onset MG post-SARS-CoV-2 infection [10,11,19,20] and post-vaccination are sparsely reported, and the pathophysiology is currently unclear [21]. The first case of post-COVID-19 new-onset MG with ocular manifestations in a 21-year-old female was reported by Huber et al. in 2020 [11]. Based on reported cases, there is no clear expected latent period between the onset of COVID-19 infection and MG symptoms. Karimi et al., in their case series, reported a 10-30-day gap between the initial COVID-19 infection and the onset of MG symptoms [22]. We performed a comprehensive review of such reported cases and summarize the findings in Table 2. On a review of the reported cases, the gap between acute COVID-19 infection and the onset of MG symptoms ranged between five days and eight weeks. In our patient, the first MG symptoms started about four weeks after acute COVID-19 infection.

**Table 2:** List of post-COVID-19 new-onset myasthenia gravis cases reported in the literature. IVIG = intravenous immunoglobulins; MG = myasthenia gravis; F = female; M = male; AChR = acetylcholinesterase receptor; MuSK = muscle-specific kinase

Serial	Author/year/reference number	Age/sex	Time of symptom onset after the COVID-19 infection	Symptoms	Thymoma	Acetylcholine receptor antibodies	Treatment received	Prognosis
1	Huber et al./2020 [11]	21/F	Two weeks	Ocular MG	No	Elevated 0.9 nmol/L	IVIG (7) (0.4 g/kg/day for five days) and oral pyridostigmine (3 × 60 mg/day)	Improved
2	Pérez Álvarez et al./2020 [23]	48/M	10 days	Ocular/binocular diplopia	No	Elevated 1.10 nmol/L	No specific MG-directed therapy given	Improved
3	Restivo et al./2020 [24]	64/M	Five days	Generalized MG/diplopia, muscle fatigability	No	Elevated 22.5 pmol/L; normal value <0.4 pmol/L	Pyridostigmine 60 mg Q six hours and prednisone 75 mg daily	Improved
4	Restivo et al./2020 [24]	71/F	Five days	Generalized MG/ocular ptosis, diplopia, and hypophonia. Later developed respiratory failure needing intubation and tracheostomy	No	Elevated 35.6 pmol/L	Plasmapheresis	Improved
5	Restivo et al./2020 [24]	68/M	Seven days	Generalized MG/muscular fatigability, diplopia, dysphagia	No	Elevated 27.6 pmol/L	IVIG 0.4 g/kg/day for five days	Improved
6	Sriwastava et al./2020 [19]	65/F	Two weeks	Ocular MG/ptosis, diplopia, fatigue	No	Elevated 7.39 nmol/L (normal<0.02 nmol/L)	Pyridostigmine. Also received IV dexamethasone for acute respiratory failure	Residual symptoms of ocular MG present on the one-month follow-up
7	Bhandarwaret al./2021 [25]	61/M	Eight weeks	Generalized MG/dysphagia, dyspnea, generalized weakness	Present (new from prior CT done at the time of the COVID-19 infection)	Elevated 11.3 nmol/L	IVIG, corticosteroids, pyridostigmine, thymectomy	Complete recovery
8	Karimi et al./2021 [22]	61/F	Six weeks	Generalized MG/dysphagia, nasal speech, ocular ptosis, diplopia, proximal muscle weakness, dyspnea	CT chest showed thymoma	Elevated 10 pmol/L; normal value, <0.4 pmol/L	Plasma exchange, pyridostigmine bromide 60 mg four times a day, and prednisone 1 mg/kg	Improved. Referred for thymoma surgery
9	Karimi et al./2021 [22]	57/M	One week	Generalized MG/general fatigue, diplopia, ptosis, and dysphagia	No	Elevated 8 pmol/L; normal value <0.4 pmol/L	Pyridostigmine 60 mg three times a day and prednisone 25 mg daily	Improved
10	Karimi et al./2021 [22]	38/F	Four weeks	Generalized MG/diplopia, ptosis, fatigue, and dysphagia	No	Elevated >8 pmol/L; normal value <0.4 pmol/L	Pyridostigmine 240 mg and prednisolone 25 mg daily were started,	Improved
11	Muralidhar Reddy et al./2021 [26]	65/M	Six weeks	Generalized MG/dysphagia	No	Elevated 4.5 nmol/L	Pyridostigmine, prednisolone, IVIG, plasma exchange, azathioprine	Improved
12	Taheri et al./2022 [10]	35/F	Three weeks	Generalized MG/severe weakness in upper/lower limbs, blurred vision, and droopy eyelids	No	Elevated 0.57 nmol/L	Pyridostigmine 60 mg three times a day	Improved
13	Assini et al./2021 [1]	77/M	Eight weeks	Bulbar MG/chewing difficulty, dysphonia, diplopia, and eyelid ptosis, worsened by muscular activity	No	Negative for AChR antibody. Positive for MuSK antibody	Pyridostigmine, azathioprine	Improved
14	Muhammed et al./2021 [27]	24/F	Four weeks	Generalized MG/bilateral fatigable ptosis, complex ophthalmoplegia, symmetrical lower motor neuron facial weakness, dysarthria. She had weakness and fatigability in all four limbs	No	Negative for AChR antibody. Positive for MuSK antibody	Pyridostigmine (60 mg four times a day) with unsatisfactory clinical response, followed by azathioprine 1.5 mg/kg/day	
15	Essajee et al./2021 [28]	7/M	Four weeks	Generalized MG/proximal muscle weakness, waddling gait, and compensatory lumbar lordosis. Fatigable bilateral ptosis	No	Elevated 0.51 nm/L anti-AChR	IV methylprednisolone, 30 mg/kg/day over three days, and IVIG, 2 g/kg, followed by 2 mg/kg/day of oral prednisone that was gradually weaned over a four-week period	Improved

Interestingly, post-infectious MG is a known entity and has been reported after Epstein-Barr virus [29], human immunodeficiency virus [30,31], viral pharyngitis [32], West Nile virus [33], and varicella [12] infections. Although the exact mechanism of post-viral MG remains unclear, molecular mimicry between the AChR and viral antigenic proteins has been speculated [32]. The latent period between COVID-19 symptoms and the onset of MG symptoms in the reported cases thus far supports this hypothesis [34]. One study in 1985 showed cross-reactivity between AChR and proteins of the bacteria *Escherichia coli*, *Proteus vulgaris*, and *Klebsiella pneumonia* [35]. Another study in 1989 showed cross-reactivity between the alpha subunit of AChR and a homologous domain on herpes simplex virus glycoprotein D [36]. The above studies have helped elucidate a possible association between infections and MG, at least in a subset of cases.

The pathogenesis of COVID-19 infection includes immune dysregulation. Like other viral infections, clinical autoimmunity may be induced due to a breakdown in immune tolerance. Liu et al. have reported multiple autoimmune antibodies that have been reported positive in patients with COVID-19 infection [37]. Other studies have also alluded to a similar theory due to the depletion of T and B cells in the setting of elevated inflammatory cytokines in patients with MG after a COVID-19 infection [24,25,27]. The most proposed mechanism for post-COVID-19 MG is molecular mimicry between SARS-CoV-2 proteins and AChR, supported by elevated acetylcholine receptor antibodies in most cases, including our case [11].

Close to 90% of MG patients have antibodies against the AChR [12]. A subset of the remaining 10% test positive for anti-MuSK antibodies. Those with anti-MuSK antibodies present with severe bulbar symptoms and respiratory failure [12]. The anti-AChR-positive MG cases are more commonly associated with other autoimmune disorders and usually have a good response to pyridostigmine. The anti-MuSK-positive MG cases are rarely associated with autoimmune disorders, pyridostigmine is less effective, and often requires steroids and steroid-sparing agents [27]. The anti-AChR antibodies are either IgG1 or IgG3 and are known to activate the complement system, thus leading to the formation of a membrane attack complex while anti-MuSK antibody is predominantly IgG4 class and not associated with complement activation [5]. Among the 16 reported cases including ours, there are only two cases that were anti-MuSK antibody positive and anti-AChR negative [1,27]. Due to the fundamental differences in the development of the two different antibodies, anti-AChR and anti-MuSK, the hypothesis of the breakdown of immune self-tolerance becomes more plausible over molecular mimicry [27].

From our literature review, there were two patients who were found to have thymoma [22,25]. In the patient reported by Karimi et al., it was postulated that the SARS-CoV-2 infection itself or the use of azithromycin may have unmasked a pre-existing latent MG [22]. Bhandarwar et al. reported an interesting case of post-COVID-19 MG where the patient developed symptoms of muscular weakness two months after COVID-19 infection and was found to have a new thymoma on CT chest imaging when compared to a CT chest from two months earlier. The patient had a complete resolution of symptoms after the thymoma was surgically resected [25]. This is the only case where there is evidence of thymoma developing after the COVID-19 infection. 

Anti-cholinesterase inhibitors are the first line for symptom management in generalized MG. However, most patients require immunosuppression directed against underlying immune dysregulation eventually during their disease course [38]. So far, there are only two reported post-COVID-19 MG cases with positive anti-MuSK antibodies, and both patients required the use of azathioprine. Our patient was positive for anti-AChR antibodies, could not tolerate pyridostigmine, but responded well to steroids. However, he became steroid dependent, and, therefore, azathioprine needed to be used as a steroid-sparing agent.

The incidence of anti-AChR-associated MG has been found to have a bimodal age of presentation [5]. The early-onset MG is described in those below 40 years of age, with 60% of cases being diagnosed between the ages of 20 and 40 years. The second peak incidence occurs after 70 years of age. The prognosis is poorer in late-onset MG. These individuals have temporary relief with the use of acetylcholinesterase inhibitor treatment, and, therefore, portend side effects with the need for long-term steroid use [39]. Interestingly, our patient had a very late presentation, with symptoms starting at the age of 82 years. To date, our patient is the oldest reported case in the literature. The temporal relation of events in our patient’s history strongly points toward a post-COVID-19 etiology of MG. Our patient had good functional capacity prior to the COVID-19 infection, making de novo MG less likely. Moreover, our patient had no personal or family history of autoimmune or musculoskeletal disease.

## Conclusions

The correct diagnosis of our patient was delayed by a few months as muscular weakness in elderly patients might be difficult to evaluate in the post-COVID-19 infection phase, especially in the presence of comorbidities. Therefore, a high index of suspicion along with a low threshold for testing is required in individuals with difficulty in weaning off ventilators. This should especially be sought out in those in whom the degree of clinical disease does not correlate with image findings not consistent with severe COVID-19 disease. It is important to remember that post-COVID-19 fatigue, long-term use of steroids, and ICU-related physical deconditioning are easy confounders and can delay the diagnosis as in our patient. As a result, we suspect that underdiagnosis of this condition is plausible which warrants increasing awareness. Further studies will be needed to help elucidate the definitive pathophysiology of this condition.
